# The Hidden Toll of Incarceration: Exploring the Link Between Incarceration Histories and Pain Among Older Adults in the United States

**DOI:** 10.1093/geroni/igad116

**Published:** 2023-10-06

**Authors:** Yulin Yang, Gabriel Lutz, Yilin Zhang, Chixiang Chen, Raya Elfadel Kheirbek

**Affiliations:** Department of Epidemiology and Biostatistics, University of California, San Francisco, San Francisco, California, USA; Department of Medicine, Division of Gerontology, Geriatrics and Palliative Medicine, University of Maryland School of Medicine, Baltimore, Maryland, USA; Department of Mathematics, University of Maryland, College Park, Maryland, USA; Division of Biostatistics and Bioinformatics, University of Maryland School of Medicine, Baltimore, Maryland, USA; Department of Medicine, Division of Gerontology, Geriatrics and Palliative Medicine, University of Maryland School of Medicine, Baltimore, Maryland, USA

**Keywords:** Incarceration, Pain, Palliative care

## Abstract

**Background and Objectives:**

Incarceration is linked to poor health outcomes across the life course. However, little is known whether and to what extent incarceration histories shape pain in later life. This study examines the relationships between incarceration histories and pain outcomes among middle-aged and older adults in the United States.

**Research Design and Methods:**

Data from a nationally representative sample of community-dwelling adults aged 51 and over in the 2012–2018 biennial waves of the U.S. Health and Retirement Study was analyzed to examine how incarceration histories influence older adults’ risks of reporting moderate-to-severe pain and pain with physical limitations. We relied on a propensity score matching approach to account for the potential confounding bias. We fit weighted generalized estimating equation models to assess the relationships between incarceration history and pain outcomes. Models were further stratified by gender.

**Results:**

After propensity score matching, our sample included 2,516 respondents aged 65 years on average (*SD* = 8.72), 21% female, and 838 with incarceration histories. Persons with incarceration histories have a greater risk of reporting moderate-to-severe pain (prevalence ratio [PR] = 1.30, 95% confidence Interval [CI]: 1.20, 1.52) and pain with physical limitations (PR = 1.48, 95% CI: 1.30, 1.68) even after adjusting for sociodemographic covariates and early life experiences. In the models stratified by gender, the associations between incarceration histories and incarceration were similar among women and men.

**Discussion and Implications:**

In a nationally representative sample of older adults (with or without incarceration history), our study demonstrates an independent association between a history of incarceration and pain in later life. Our findings highlight the far-reaching impact of incarceration and the need for developing optimal management strategies to reduce the burden of disabling pain. Interventions should prioritize socioeconomically vulnerable groups who may have the least access to pain treatment in later life.

**Translational Significance:** The relationship between incarceration and chronic pain in community-dwelling older adults has significant implications for clinical practice and policy development. This evidence can inform healthcare professionals to consider a patient’s incarceration history and how it may impact the development of chronic pain and overall well-being. Patients with a history of incarceration may require specialized care and treatment, including tailored pain management strategies that account for their unique experiences and needs. Using population-based data to explore the connection between incarceration and chronic pain can provide valuable insights for policymakers and public health advocates who are advocating for alternatives to incarceration.

The American penal system has seen an unprecedented expansion in the past five decades, with the incarceration rate increasing approximately five-fold since the mid-1970s, peaking in 2008 (Kluckow & Zeng, 2022; Pettit & Western, 2004; The Sentencing Project, 2023). Each day, about two million individuals find themselves under some form of penal control, with two-thirds in state or federal prisons for longer sentences and one-third in local or federal jails for shorter terms (Maruschak & Minton, 2020; Sawyer & Wagner, 2023). Over six million U.S. residents have experienced or currently experience incarceration (Massoglia & Pridemore, 2015; Sawyer & Wagner, 2023). The consequences of the mass incarceration are profoundly harmful because one’s life trajectory can be deeply affected by having a criminal record (Pettit & Western, 2004).

Incarceration is a significant public health concern with detrimental effects on the health of both currently and formerly incarcerated individuals (Dumont et al., 2012; Massoglia & Pridemore, 2015; Massoglia & Remster, 2019; Wildeman & Wang, 2017). Those with a history of incarceration face an elevated risk for chronic health problems (Howell et al., 2016; Schnittker & John, 2007; Wang et al., 2009), mental health disorders (Schnittker, 2014), infectious diseases (Massoglia, 2008), and weight gain (Clarke & Waring, 2012; Houle, 2014) compared with non-incarcerated individuals. Research documents an increased risk of mortality immediately after release and in the years following incarceration (Binswanger et al., 2007, 2011; Massoglia et al., 2014; Rosen et al., 2008; Spaulding et al., 2011). Leading causes of mortality include drug overdose, suicide, and homicide (Binswanger et al., 2011; Bukten & Stavseth, 2021; Massoglia et al., 2014), as well as other negative effects such as suicidal thoughts, self-injury, violent behaviors, and psychological vulnerability (Borschmann et al., 2017; Wildeman & Andersen, 2020). Some studies have noted variations in the incarceration-mortality link based on race and gender (Massoglia et al., 2014; Patterson, 2010). Additional evidence suggests a protective effect on mortality among young Black men due to access to basic healthcare within confinement, resulting in fewer accidental deaths compared to the community (Massoglia et al., 2014).

The aforementioned studies have contributed to our understanding of the relationship between incarceration and health, but they primarily focus on young or middle-aged individuals. Limited knowledge exists regarding the health impact of incarceration in later life. The share of older adults with a history of incarceration has steeply risen (Williams et al., 2021), and projections suggest that by 2030, individuals over 55 will constitute one-third of the U.S. prison population (Skarupski et al., 2018). Many of today’s older adults, particularly those from the Baby Boomer generation born between 1946 and 1964, experienced the era of mass incarceration during in their early or mid-adulthood (Latham-Mintus et al., 2022). This is especially true for minoritized older populations, as mass incarceration has resulted from decades of strict crime policies, controversial police practices, and racially biased sentencing laws (Garland, 2001; Maschi et al., 2017; Wildeman & Wang, 2017; Williams et al., 2021). Indeed, due to mass incarceration, Pettit and Western (2004) argue that incarceration, akin to education, has become a common life event for young Black men from specific birth cohorts with lower levels of education, and has profound consequences including health outcomes across their life course.

Understanding the impact of incarceration on health over the life course is crucial, especially as individuals age. Currently or formerly incarcerated older adults are more likely to experience cognitive and functional impairment, as well as other geriatric syndromes, at a relatively young age—a phenomenon referred to as “accelerating aging” (Williams et al., 2012, 2021). A nascence of studies have examined and revealed that a history of incarceration is associated with unsuccessful aging and poor health outcomes in later life such as more functional limitations, worse mental health outcomes, cognitive impairment, and other health problems than those without such a history (Cox & Wallace, 2022; Garcia-Grossman et al., 2023; Latham-Mintus et al., 2022; Pruchno & Wilson-Genderson, 2015; Williams et al., 2010).

Although one study found that currently incarcerated older adults in jail settings have a disproportionately high prevalence of severe and frequent pain (Williams et al., 2014), there is a surprising lack of research on pain among formerly incarcerated individuals. This is concerning because untreated pain can be a significant contributing factor to substance abuse and addiction (e.g., opioids overdose), which in turn may lead to further incarcerations due to drug-related offenses. This is particularly relevant given the opioid epidemic and high rates of reoffending.

There are several underlying mechanisms linking incarceration histories to pain in later life. First, being incarcerated can involve physical traumas, such as violence and exposure to dangerous environments, leading to injuries and chronic pain that persist even after release (Piper & Berle, 2019). Moreover, the experience of incarceration often causes high levels of psychological stress, including trauma, stigma, and social isolation (Western et al., 2015), which can contribute to the development and exacerbation of pain conditions (Abdallah & Geha, 2017). Incarceration histories can also be associated with various midlife health problems, such as hypertension, obesity, and infectious diseases (Massoglia & Remster, 2019), which can collectively contribute to pain in later life. Fourthly, incarcerated people with chronic conditions were often released without medications and follow-up appointments in the community at release and often lacked health insurance, which could lead to untreated or poorly managed medical conditions (e.g., arthritis), including chronic pain (Wildeman & Wang, 2017). Lastly, reintegration challenges faced by formerly incarcerated individuals, such as unstable housing or homelessness, unemployment, or discrimination in employment, poverty, and limited social support networks, can also contribute to pain as they age (Massoglia & Remster, 2019; Wildeman & Wang, 2017; Williams et al., 2010).

Notably, these mechanisms might interact with each other to affect pain and overall health as older adults with incarceration histories often face multiple risk factors simultaneously. These findings highlight the need for further research into the specific health concerns faced by older adults with a history of incarceration and the development of targeted interventions to address these issues.

Despite the evidence showing potential pathways between incarceration and pain, little is known about whether and how incarceration histories shape chronic pain outcomes in later life. This can be attributed to the fact that data on incarceration is often fragmented across different correctional institutions at the county, state, and federal levels (Wang et al., 2019). Additionally, there is a scarcity of national population-based surveys that inquire about high-quality health indicators and measures of incarceration (Ahalt et al., 2012). In addition to data limitation, establishing causal connections between incarceration and pain (as well as other health outcomes) is also challenging due to selection issues. Specifically, structural factors (e.g., poverty, low levels of education), childhood experience, and behavioral risk factors (e.g., drug use or exposure to violence) can be associated with both the risk of incarceration and poor health outcomes (Massoglia & Remster, 2019).

Taken together, this study evaluates the associations between experiencing incarceration and pain outcomes in a nationally representative sample of individuals aged 51 years and older. Here we refer to people aged 51–64 years (together with the rest of the sample aged 65 years and above) as older adults, considering the aforementioned evidence suggesting that currently or formerly incarcerated individuals are more likely to undergo accelerated aging. We expect that older adults with a history of incarceration are more likely to experience moderate-to-severe pain and pain with physical limitations. This is attributed to the cumulative risk of incarceration that individuals face from early or midlife until they reach their fifties or older.

Moreover, the population with incarceration histories differs significantly from the general population. Although men make up the majority of incarcerated individuals (Federal Bureau of Prisons, 2023), there has been a notable increase in the number of women, especially women of color, with incarceration histories (Sawyer, 2018). People of color, particularly Black men, experience disproportionately higher rates of incarceration and receive harsher sentences compared to their White counterparts (Boen et al., 2022; Tucker Sr, 2014). To address potential selection bias, we employed a propensity score matching approach to create a balanced sample. Then we evaluated the prevalence of pain outcomes over 6 years among respondents with similar propensity of being incarcerated, then stratified by men and women. Notably, our findings can only be generalized to formerly incarcerated individuals and those with similar backgrounds who do not have a history of incarceration.

## Data and Methods

### Study Design and Population

We conducted a secondary analysis using data from four survey waves of the U.S. Health and Retirement Study (HRS, 2012–2018). The HRS is a nationally representative ongoing survey of community-dwelling adults aged over 51 years and their spouses (of any age) conducted by the University of Michigan and sponsored by the National Institute of Aging. The core interviews take place every 2 years and collect comprehensive information on respondents’ health, including pain, as well as their income, income, wealth, employment, family connections, and psychosocial and lifestyle factors (Servais, 2010). The HRS has been identified as being “uniquely positioned to address many priority areas of palliative care research” (Kelley et al., 2014). Additionally, in alternating waves, the HRS collects information on psychosocial and lifestyle factors through self-administered questionnaires known as Leave-Behind Questionnaires (LBQ), which are completed by a random half of the sample (Smith et al., 2023).

In 2012 and 2014 LBQs, the HRS included an experimental section called “Unusual Living Conditions,” which specifically asked about incarceration histories and other life experiences such as being homeless and unfair treatment in everyday life (e.g., at work, applying for bank loans). For this study, we included all respondents who completed this experimental module. We then followed up with these respondents every 2 years through the core survey until 2018 to gather information on their pain status. The analysis was conducted using publicly available de-identified data, which did not require institutional review board review. Our findings are reported following the Strengthening the Reporting of Observational Studies in Epidemiology (STROBE) reporting guideline (Von Elm et al., 2014).

Among the 24,628 respondents in the 2012 HRS core interview and 23,224 respondents in the 2014 HRS core interview, a total of 10,079 participants were eligible for the 2012 LBQ, and 9,549 were eligible for the 2014 LBQ. To maximize our sample size, we combined the two LBQ subsamples, resulting in a total of 19,628 respondents who were eligible to participate in the 2012 or 2014 LBQs. We first excluded 4,681 respondents who did not complete and/or return the LBQs. Additionally, we excluded 435 respondents who were younger than 51 years at baseline because the HRS is nationally representative for individuals aged 51 and older. Next, we excluded 469 respondents who did not answer the question about their history of incarceration. Notably, those respondents who were excluded due to missing information on incarceration were older (70 vs 68 years), more likely to be non-Hispanic Black (24% vs 16%) or Hispanic/Latinx (17% vs 11%), less likely to have a high school degree (31% vs 16%), not married (49% vs. 41%), experienced childhood poverty (35% vs 29%), and had poorer childhood health (3% vs 1%) compared to their included counterparts. Additionally, 75 respondents were excluded due to missing pain information at baseline. We also excluded individuals with missing data on socio-demographic covariates (*n* = 122) and childhood adverse experiences (*n* = 1,452). Respondents with a missing value for childhood adverse experiences were younger, with a history of incarceration, individuals of color, not currently married, and without a high school degree compared with those without the missing value. To address potential bias from extreme survey weights (i.e., weights of 0 or larger than 30,000), we removed respondents with weights of 0 and adjusted the weights of observations with weights larger than 30,000 to a value of exactly 30,000.

Following prior studies (e.g., Garcia-Grossman et al., 2023; Steigerwald et al., 2022), we conducted our analysis using data from complete cases by excluding the individuals with missingness for included variables. After excluding individuals with missing data on pain outcomes and covariates, our pre-match analytic sample included 838 respondents who reported a history of incarceration and 11,136 respondents without an incarceration history. In our sample, more than 97% of respondents reported pain twice over the study period with less than 3% only reporting pain once (i.e., only reported pain at baseline). After propensity score matching (1:2 ratio), our final sample consisted of 1,676 respondents without an incarceration history who were matched to the 838 respondents with an incarceration history, based on similar sociodemographic characteristics and childhood experiences (see Supplementary Figure 1).

### Measures

#### Pain outcomes

Experiences with pain were assessed consistently in all waves. The survey asked respondents “*Are you often troubled with pain?*” If respondents answered affirmatively, they were then asked, “*How bad is the pain most of the time: mild, moderate or severe?*” and “*Does the pain make it difficult for you to do your usual activities such as household chores or work?*” The first measured pain outcome is a dichotomous variable of pain severity—moderate-to-severe pain—that was recoded from the first two questions (1 = moderate or severe pain vs 0 = no pain or mild pain). The second measured pain outcome is a dichotomous variable of pain interferences—pain with physical limitation—that was coded from the first and third questions (1 = pain limits usual activities vs 0 = no pain or pain does not limit usual activities).

#### Self-reported incarceration history

In 2012 or 2014 LB questionnaires, respondents were asked “*Have you ever been an inmate in a jail, prison, juvenile detention center, or other correctional facility?*” Those who answered affirmatively were coded as having incarceration history (IH); those who answered negatively were coded as having no IH.

#### Sociodemographic characteristics

This study controlled for sociodemographic characteristics such as age (years), female gender (vs male gender as the reference group), race and ethnicity (non-Hispanic White as reference group, non-Hispanic Black, Latino/Hispanic, and non-Hispanic Other), educational attainment (no degree as the reference group, high school degree/GED, some college, and four-year college degree and above), marital status at baseline (married as reference group, separated or divorced, widowed, and never married).

#### Childhood experiences

Following prior research (Latham-Mintus et al., 2022), this study controlled for childhood experiences because they could be potential confounders for pain outcomes and a risk of being formerly incarcerated (Goosby, 2013; Roos et al., 2016). Childhood socioeconomic status (SES) was assessed based on the question “*Now think about your family when you were growing up, from birth to age 16. Would you say your family during that time was pretty well off financially, about average, or poor?*” Responses were coded as a binary variable (poor vs well off financially or about average). Childhood health was assessed based on the question “*Consider your health while you were growing up, before you were 16 years old. Would you say that your health during that time was excellent, very good, good, fair, or poor?*” Responses were coded as a binary variable (poor childhood health vs excellent, very good, good, or fair). Living with a grandparent was assessed based on the question “*Did you ever live in the same household with a grandparent for a year or more before age 17?*” (yes vs no).

We also included a scale measuring adverse childhood experiences with four items that happened before 18 years old: had to do a year of school over again, ever in trouble with the police, parents drank or used drugs so often that it caused problems in the family, and ever physically abused by either of parents. This information was available in the Aggregated HRS Childhood Family and Health database (Zhang et al., 2020).

### Analytical Strategy

**Figure 1.**
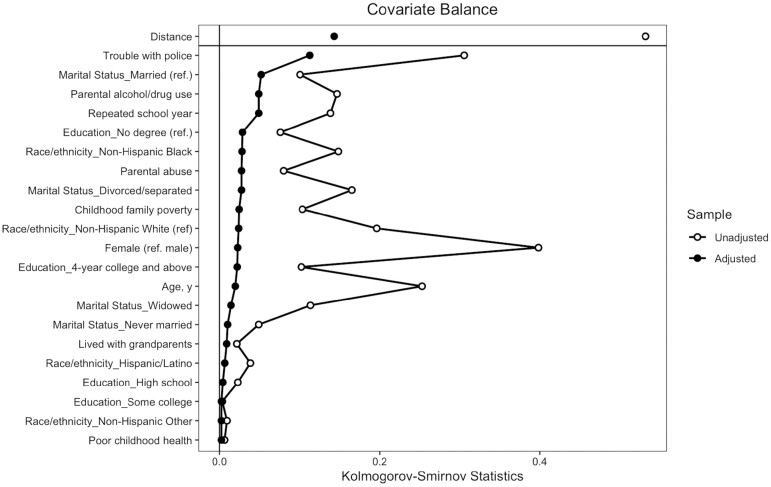
Love plot displaying Kolmogorov–Smirnov Statistics comparing sociodemographic characteristics and childhood adverse experience between older adults with or without incarceration histories before (unadjusted) and after (adjusted) propensity score matching.

People with incarceration histories might be highly selective compared to the general population, thus, to make a fair comparison, we need to compare those with incarceration histories to the control groups with similar characteristics but who had not been formerly incarcerated. Indeed, if the two groups were very different, using a regression adjustment approach without matching can increase bias in the estimated treatment effects (Stuart, 2010). Although randomized assignment is widely considered to be a “golden standard” in experimental design, it cannot be applied directly in observational research. Therefore, we use propensity score matching to minimize the effects of confounding variables when randomized assignment is not possible or practical (Austin, 2011; Ho et al., 2007). In particular, we performed multiple matching approaches such as logistic regression and random forest with a matching ratio 1:2 or 1:3. Following prior research on incarceration and health (Massoglia, 2008), we included several covariates in the process of matching with propensity score approach to get a balanced sample: age, gender, race/ethnicity, education, marital status, early life conditions (i.e., family poverty, live with grandparents, and poor childhood health), and adverse childhood experience (i.e., repeated school year, trouble with police, parent drinking or drug problems, or abused by parents). After comparing matching effects among all the approaches, the logistic regression with a ratio 1:2 provided the best matching performance (see Figure 1). The sample characteristics between matched and unmatched samples are summarized in Table 1.

**Table 1.** Descriptive Characteristics of Older Adults by History of Incarceration, Before And After Propensity Score Matching (Health and Retirement Study, 2012/2014)

Characteristics	Before propensity score matching (*n* = 11,974)			After propensity score matching (*n* = 2,514)		
	Ever incarcerated		*p*-Value	Ever incarcerated		*p*-Value
	No (*n* = 11,136)	Yes (*n* = 838)		No (*n* = 1,676)	Yes (*n* = 838)	
	*n* (%) or mean (±*SD*)	*n* (%) or mean (±*SD*)		*n* (%) or mean (±*SD*)	*n* (%) or mean (±*SD*)	
Age, years	69.37 (±10.1)	63.49 (±8.7)	.000	64.07 (±8.7)	63.49 (±8.7)	.750
Female (ref. male)	6,907 (62.0)	186 (22.2)	.000	334 (19.9)	186 (22.2)	.190
Race/ethnicity			.000			.470
Non-Hispanic White (ref)	8,151 (73.2)	449 (53.6)		938 (56.0)	449 (53.6)	
Non-Hispanic Black	1,590 (14.3)	244 (29.1)		441 (26.3)	244 (29.1)	
Hispanic/Latino	1,100 (9.8)	115 (13.7)		241 (14.4)	115 (13.7)	
Non-Hispanic other	259 (2.7)	30 (3.6)		56 (3.3)	30 (3.6)	
Education			.000			.270
No degree (ref.)	1,654 (14.9)	188 (22.4)		328 (19.6)	188 (22.4)	
High school	6,031 (54.2)	473 (56.4)		953 (56.9)	473 (56.4)	
Some college	652 (5.9)	52 (6.2)		108 (6.4)	52 (6.2)	
4-year college and above	2,799 (25.1)	125 (14.9)		287 (17.1)	125 (14.9)	
Marital status			.000			.100
Married (ref.)	6,647 (59.7)	416 (49.6)		919 (54.8)	416 (49.6)	
Divorced/separated	1,695 (15.2)	266 (31.7)		486 (29.0)	266 (31.7)	
Widowed	2,315 (20.8)	79 (9.4)		134 (8.0)	79 (9.4)	
Never married	479 (4.3)	77 (9.2)		137 (8.2)	77 (9.2)	
Early-life conditions						
Family poverty	3,179 (28.6)	326 (38.9)	.000	611 (36.5)	326 (38.9)	.240
Lived with grandparents	2,856 (25.7)	233 (27.8)	.169	451 (26.9)	233 (27.8)	.630
Poor childhood health	142 (1.3)	16 (1.9)	.121	36 (2.1)	16 (1.9)	.770
Adverse childhood experience						
Repeated school year	1,620 (14.6)	238 (28.4)	.000	394 (23.5)	238 (28.4)	.008
Trouble with police	479 (4.3)	292 (34.8)	.000	395 (23.6)	292 (34.8)	.000
Parental alcohol/drug use	1,821 (16.4)	260 (31.0)	.000	438 (26.1)	260 (31.0)	.010
Parental abuse	811 (7.3)	128 (15.3)	.000	210 (12.5)	128 (15.3)	.060
Moderate-to-severe pain at baseline	2,820 (25.3)	292 (34.8)	.000	427 (25.5)	292 (34.8)	.000
Pain with physical limitations at baseline	2,477 (22.3)	303 (36.2)	.000	381 (22.8)	303 (36.2)	.000

We assessed the prevalence ratios of moderate-to-severe pain and pain with physical limitations based on incarceration histories. In analyzing the prevalence of pain with physical limitations, we chose not to exclusively focus on individuals with pain for several reasons. Firstly, we considered pain with limitations as an indicator of pain’s impact on daily life, assuming that pain without limitations has minimal effects on daily activities. Secondly, due to limited statistical power in our sample, we did not restrict the analysis to respondents with pain alone, given the small sample size of 838 individuals with a history of incarceration. Lastly, in our longitudinal analysis aiming to capture the trajectory of pain with limitations, restricting the analytical sample based on wave-specific pain status could complicate result interpretation.

To estimate the longitudinal prevalence ratios of pain outcomes we fitted weighted generalized estimating equations (WGEE) with Poisson distribution and AR1 structure to quantify within-patient correlation (Barros & Hirakata, 2003; Robins et al., 1995). In each model with post-matching data, we included gender and race/ethnicity controlled for three adverse childhood experience variables (i.e., trouble with the police, repeated school year, and parental alcohol/drug use) that were not well matched to account for the selection bias (see Table 1 right column). We also included the time fix effects to account for the time-related variability in the data. The WGEE models can address this potential bias due to imbalanced data among respondents, as we considered both sampling weight and probability of missingness to account for complex survey design and attrition. This method assumes that the missing mechanism is either missing completely at random or missing at random (MAR; Chen et al., 2019; Shardell & Miller, 2008). MAR is a type of missing data mechanism where the missingness is not random but can be fully accounted for by completely observed variables. We further tested gender and racial/ethnic variations of the relationships between incarceration and pain outcomes using multiplicative interaction. To check the robustness of our analysis to the presence of unmeasured confounders, we also conducted sensitivity analysis by calculating Rosenbaum’s bounds (Rosenbaum, 1987). Analyses were carried out in R version 4.1.0.

## Results

### Descriptive Characteristics

In the original sample before matching (see Table 1, left column), the average baseline age of our respondents was 64 years old for those who were formerly incarcerated and 69 years old for those who were not. Among those previously incarcerated, women comprised 22% of respondents, versus comprising 62% of respondents without IH. Moreover, the percentage of non-Hispanic Black respondents with IH was disproportionately high (29.1% with vs 14.5% without), compared with that of their non-Hispanic white counterparts (53.6% with vs 73.2% without). People with IH were more likely to report adverse childhood experiences and lower childhood SES; 38.9% of respondents with IH reported family being poor during childhood (vs 28.6% without IH). The percentage of all four childhood adverse experience indicators was disproportionally high among those with IH; 28.4% of respondents with IH repeated school (vs 14.6% without), 34.8% of respondents with IH had trouble with the police (vs 4.3% without), 31% of respondents had parents with alcohol/drug problems (vs 16% without) and 15.3% of respondents with IH were abused by parents (vs 7.3% without). At baseline, there were 34.8% respondents with IH that reported moderate-to-severe pain (vs. 25.3% without IH) and 36.2% that reported pain with physical limitation (vs. 22.3% without IH). The sample characteristics after matching are shown in Table 1 (right column), and the comparison of the sample characteristics before and after matching is shown in Figure 1. After performing the matching process, we achieved a balanced sample in most variables; however, there was still an imbalance in the variable assessing experiences of trouble with the police before the age of 18. Therefore, we controlled this variable in each GEE model.

### Prevalence Ratios: Moderate-to-Severe Pain and Pain With Physical Limitations

The results of WGEE models for the prevalence ratio of *moderate-to-severe pain* controlling for experiences of trouble with the police before the age of 18 and time-fixed effects are presented in Table 2. Among 2,514 matched older adults, those with IH had a higher prevalence of reporting moderate-to-severe pain (prevalence ratio [PR]: 1.35, 95% confidence interval [CI]: 1.20, 1.52) in Model 1. Compared with men, women had a higher prevalence of reporting moderate-to-severe pain (PR: 1.47, 95% CI: 1.29, 1.68). In Models 2 and 3, we present results stratified by women and men. The prevalence of moderate-to-severe pain is higher for both women (PR: 1.35, 95% CI: 1.08, 1.67) and men (PR: 1.36, 95% CI: 1.18, 1.57) with IH compared to their counterparts without IH. The 95% CIs for men and women overlap but the interval is narrower for men due to their larger sample size of men. We conducted formal tests of gender differences by incorporating multiplicative interaction terms, and the results were not statistically significant. In Model 2, we found that the prevalence of moderate-to-severe pain is higher for non-Hispanic Black women (PR: 1.47, 95% CI: 0.84, 1.30) compared with non-Hispanic White women. However, due to the small sample size, the 95% CI crosses the null. Similarly, in Model 5, we observed that the prevalence of moderate-to-severe pain is higher among men self-identified as non-Hispanic other races (PR: 1.46, 95% CI: 1.07, 2.00) compared to non-Hispanic White men. However, we did not observe similar findings among women. This suggests that both women and men, with similar propensities for being formerly incarcerated, experienced negative effects of incarceration histories on pain outcomes.

**Table 2.** Weighted Generalized Estimating Equations (WGEE) Models of Moderate-to-Severe Pain Using Post-Matching Sample (Health and Retirement Study 2012–2018; *n* = 2,514)

Variable	Model 1 Women + men		Model 2 Women		Model 3 Men	
	PR	95% CI	PR	95% CI	PR	95% CI
Ever incarcerated	1.35***	1.20, 1.52	1.35***	1.08, 1.67	1.36***	1.18, 1.57
Female (ref. male)	1.47***	1.29, 1.68				
Race/ethnicity (ref. Non-Hispanic White)						
Non-Hispanic Black	0.97	0.84, 1.11	1.47	0.84, 1.30	0.93	0.78, 1.11
Hispanic/Latino/a	0.96	0.79, 1.17	0.97	0.69, 1.48	0.95	0.76, 1.19
Non-Hispanic other	1.35*	1.02, 1.79	0.96	0.67, 1.95	1.46*	1.07, 2.00

*Notes:* CI = confidence interval; PR = prevalence ratio. All models are controlled for trouble with police before age 18 and time fixed effects. All models fit weighted generalized estimating equations (WGEE).

*** *p* < .001, ** *p* < .01, * *p* < .05, † *p* < .10.

The results of WGEE models for the prevalence ratio of *pain with physical limitations* controlling for experiences of trouble with the police before the age of 18 and time-fixed effects are presented in Table 3. In Model 4, Older adults with IH had a higher prevalence of reporting pain with physical limitations (PR: 1.48, 95% CI: 1.30, 1.68). Women had a higher prevalence of reporting pain with physical limitations compared with men (PR: 1.47, 95% CI: 1.27, 1.70). When stratified by gender, both women (see Model 5; PR: 1.47, 95% CI: 1.15, 1.88) and men (see Model 6; PR: 1.49, 95% CI: 1.29, 1.73) with incarceration histories had a higher prevalence of pain with physical limitations compared with their counterparts without incarceration histories. The CIs for men and women overlap, but the interval is narrower for men due to their larger sample size. The multiplicative interaction terms did not reach statistical significance. In Model 6, the results were consistent with those in Model 3, indicating that men who self-identified as another race had a higher prevalence of pain with physical limitations compared to non-Hispanic White men. These findings align with the results of the models assessing the prevalence of moderate-to-severe pain, suggesting that a history of incarceration was associated with higher levels of pain with limitations for both women and men who had similar propensities for being formerly incarcerated.

**Table 3.** Weighted Generalized Estimating Equations (WGEE) Models of Pain With Physical Limitations Using Post-matching Sample (Health and Retirement Study 2012–2018; *n* = 2,514)

Variable	Model 4 Women + men		Model 5 Women		Model 6 Men	
	PR	95% CI	PR	95% CI	PR	95% CI
Ever incarcerated	1.48***	1.30, 1.68	1.47**	1.15, 1.88	1.49***	1.29, 1.73
Female (ref. male)	1.47***	1.27, 1.70				
Race/ethnicity (ref. Non-Hispanic White)						
Non-Hispanic Black	1.02	0.88, 1.18	1.02	0.80, 1.30	1.01	0.83, 1.22
Hispanic/Latino/a	1.06	0.88, 1.29	1.05	0.68, 1.60	1.07	0.86, 1.33
Non-Hispanic other	1.50**	1.13, 1.99	1.03	0.55, 1.95	1.74**	1.31, 2.32

*Notes*: CI = confidence interval, PR = prevalence ratio. All models are controlled for trouble with police before age 18 and time fixed effects. All models fit weighted generalized estimating equations (WGEE).

*** *p* < .001, ** *p* < .01, * *p* < .05, † *p* < .10.

### Sensitivity Analysis

#### Gamma statistics

Given that the two primary outcomes were dichotomous variables, we conducted a sensitivity analysis to assess the impact of unobserved confounding on the treatment effect estimate. Supplementary Figure 2 shows the gamma statistic is 1.20 for moderate-to-severe pain, indicating that the odds of receiving treatment attributed to unmeasured confounders can be up to 1.2 times between treated and control groups such that the Rosenbaum bounds contain *p*-values smaller than .05 (Rosenbaum, 1987). A Gamma value of 1.2 is relatively small, thus the impact of unobserved confounding is moderate to strong. And for pain with physical limitations, the Gamma statistic is 1.45, indicating the impact of unobserved confounding is mild. This suggests that the treatment effect estimate is likely to be robust to unobserved confounding, and the study findings may be considered more reliable.

We conducted several sensitivity analyses to show the robustness of our findings. First, we used prematching full sample, and the results were almost identical with slightly larger effect sizes (see Supplementary Table 2). Second, we used data with one-to-one matching (*n* = 1,676), and the results were almost identical to the results presented in Tables 2 and 3 (see Supplementary Table 2).

## Discussion

This study examines the impact of incarceration on pain outcomes among older adults. Using data from a nationally representative sample of older adults and employing propensity score matching, we found that formerly incarcerated older adults report higher rates of moderate-to-severe pain and pain with physical limitations compared to their non-incarcerated counterparts with a similar propensity for incarceration. To our knowledge, this is the first study that used population-based data and leverage propensity score methods, to study the associations between incarceration histories and prevalence of pain and pain-related limitations.

This study contributes to the current understanding of the effects of incarceration on health outcomes in later life by suggesting the relationship is causal using propensity score matching. Thus far, prior studies utilizing the HRS and other data sets found formerly incarcerated older adults have significantly worse health outcomes and a higher risk of impairment (Cox & Wallace, 2022; Garcia-Grossman et al., 2023; Latham-Mintus et al., 2022). However, little is known whether these established associations were due to confounding bias. Indeed, analyses from Cox and Wallace (2022) show that after adjusting for socioeconomic status (SES) and adverse childhood experience (ACE), the effect of incarceration on cognitive function and cognitive impairment went from statistically significant to insignificant, indicating SES and ACE could be the confounders for the risk of incarceration and poor cognitive function.

Our analysis employed propensity score matching to account for several potential confounders, including individual socio-demographic characteristics and early life experiences, which were identified in previous studies (Massoglia, 2008; Massoglia et al., 2014) and available in the HRS data set. We matched incarcerated individuals with a control group of nonincarcerated respondents who had similar backgrounds. However, because the timing and reasons for incarceration were not available in the HRS data set, we did not include other variables that could potentially serve as both confounders and mediators, such as employment status, household wealth, chronic conditions, and mental health histories. Our analyses found that older adults with a history of incarceration exhibited a higher prevalence of moderate-to-severe pain and pain with physical limitations. It is important to note that the propensity score matching method resulted in significant changes in the composition of the nonincarcerated group, with a majority of males and non-Hispanic Black and Hispanic/Latino/a individuals. Therefore, the generalizability of our findings is limited to this specific population rather than the broader population of non-incarcerated individuals.

In the gender-specific WGEE models, we observed that a history of incarceration was associated with a higher prevalence of moderate-to-severe pain and pain with limitations for both women and men who had similar propensities for being formerly incarcerated. These findings suggest that the effects of incarceration on pain outcomes were similarly detrimental across all gender and racial/ethnic subgroups with similar backgrounds. Our findings differ from recently published reports (Latham-Mintus et al., 2022) which reported that older women with incarceration histories reported higher levels of functional limitation; however, after propensity score matching the sample composition of our study was found to be different from that from Latham-Mintus et al. (2022). Moreover, the nonincarceration sample size was significantly dropped, particularly for women, and thus the statistical power may limit our analyses to detect significant variations across different subgroups. Furthermore, these null results are meaningful and suggest that older adults with incarceration histories may be a highly selective subpopulation.

There are several underlying causal pathways related to SES and material factors, behavioral factors, and psychological factors that link incarceration to pain among older adults. First, incarceration in early or mid-life significantly hinders one’s chances of completing an education, which disqualifies them from many specialized careers. Second, possession of a criminal record predisposes individuals to widespread hiring biases making employment more difficult to obtain, further hindering their chances for economic growth. Third, a history of incarceration poses a significant cumulative challenge to personal, social, and economic growth, which likely leads to reduced health literacy and reduced ability to obtain health insurance and maintain regular medical care with a primary provider.

These SES and/or material factors caused by incarceration can also lead to behavioral risk factors regarding pain outcomes. For example, research found that factors such as lack of access to healthcare, poor nutrition, substance use, and related addiction are more prevalent among individuals with a history of incarceration (McNiel et al., 2005; Wang et al., 2019). Substance use disorders can contribute to physical health issues and may exacerbate pain symptoms (Savage et al., 2008).

In addition to material and behavioral factors, psychological factors are important to link incarceration experience to pain outcomes. Former incarceration may cause significant repercussions on an individual’s mental health due to experiences of trauma, violence, and chronic stress associated with living in adverse conditions (Schnittker, 2014). Individuals with IH may find it challenging to reintegrate into society causing further stress and emotional anguish.

Geography and policy landscape, such as criminalization of drug use and variations in law enforcement practices, can influence the connection between incarceration histories and later-life pain outcomes. Social support programs and healthcare access play significant roles in postincarceration outcomes. Social welfare policies provide resources for successful reintegration (Western et al., 2015), while healthcare—including Medicaid coverage—addresses medical and mental health needs (Howell et al., 2022). Policy variations affect re-entry programs, vocational training, and housing assistance (Travis, 2005). Implementation and accessibility of these policies impact an individual’s well-being during reintegration. Understanding the relationship between incarceration histories and health outcomes in older adults requires considering contextual factors. Future research should delve into social benefits, Medicaid/health insurance availability, and policy-related elements to better comprehend their influence on the re-acclimation process after incarceration.

This study underscores the significant association between a history of incarceration and poor health outcomes in older adults. Health care professionals and policymakers need to consider the health implications of incarceration for both recently released older adults and those with a remote history of incarceration (Jahn, 2020). Population-based health studies must collect detailed information about criminal legal involvement to further elucidate the health impacts of incarceration over the life course. Future research should explore the temporal relationships between incarceration events and health outcomes, the role of recurrent incarceration events, and the effects of long-term incarceration on older adults’ health and well-being. Given that incarceration is more prevalent among racial and ethnic minority individuals, additional research is necessary to evaluate its contribution to health disparities. This study highlights the importance of screening for incarceration in primary care settings and the need for healthcare professionals to receive enhanced training about the effects of incarceration on individuals and communities. Public health and healthcare professionals should prioritize interventions that mitigate the long-term poor health outcomes present among people with incarceration history. Understanding the long-term consequences of incarceration on community health is crucial as policymakers consider ways to reduce the footprint of mass incarceration in the United States.

## Limitations

We acknowledge several limitations that should be considered when interpreting our study findings. Although the HRS is one of the few population-based surveys that inquire about incarceration histories among older adults, the question used may not be adequate for capturing the complexities of the relationship between incarceration and health disparities. In particular, more detailed information regarding incarceration histories, such as the frequency of incarceration and the timing or life stage of incarceration, was not available in HRS data sets. Previous research has indicated that an increased frequency of incarceration is associated with higher mortality risk (Hawks et al., 2020), and that incarceration during early life is more detrimental to health compared with later stages of adulthood (Baćak et al., 2019). The HRS did not ask about reasons for incarceration, such as drug-related crimes; thus, we cannot rule out the possibility, for example, that those who were incarcerated were addicted to opioids due to prior pain status. Moreover, this study did not control for the duration of incarceration as findings from several studies suggest the experience of incarceration as an exposure generally has a greater effect on health than the length of incarceration (Massoglia, 2008; Schnittker & John, 2007). Future research may consider using data with more detailed incarceration measures to test the reliability of our findings. Second, the HRS pain questions do not assess these different dimensions of pain such as specifying the pain’s location or cause, comorbidities, or its functional or psychological impact. Lacking such measures, this study can only rely on a general assessment of pain but not specific dimensions such as pain intensity, interference, or quality. Third, although in calculating propensity scores, we have controlled a variety of variables that have been identified in the prior study by Massoglia (2008), we are not able to include all potential confounders—such as drug use, mental health, pain status before and during incarceration, and other variables—that Massoglia (2008) used in their propensity score analysis. Therefore, it is possible that the associations between incarceration and poor pain outcomes are the result of unaccounted confounders; however, we have applied multiple methods to account for the confounding bias such as applying propensity score matching, using inverse probability weighting, and survey weights. Lastly, both pain outcomes and incarceration (i.e., exposure) in this study are self-reported, thus we cannot rule out that the possibility of the same source bias. Some factors, such as psychological disposition, could influence respondents’ reporting on their incarceration and pain outcomes.

## Conclusion

The relationship between incarceration and chronic pain in older adults living in the community has significant implications for clinical practice and policy development. This evidence can inform healthcare professionals to consider a patient’s history of incarceration and how it may impact their overall health and well-being, including the development of chronic pain. Patients with a history of incarceration may require specialized care and treatment, including tailored pain management strategies that account for their unique experiences and needs. Additionally, using population-based data to explore the connection between incarceration and chronic pain can provide valuable insights for policymakers and public health advocates who are advocating for alternatives to incarceration.

## Supplementary Material

igad116_suppl_Supplementary_Figures_S1-S2_Tables_S1-S2
