# Supplementary material for: The Hidden Toll of Incarceration: Exploring the Link Between Incarceration Histories and Pain Among Older Adults in the United States
Source: Innov Aging. 2023 Oct 6;7(10):igad116. doi: 10.1093/geroni/igad116 (PMC10714910; doi:10.1093/geroni/igad116)
Supplement: igad116_suppl_Supplementary_Figures_S1-S2_Tables_S1-S2 [file igad116_suppl_supplementary_figures_s1-s2_tables_s1-s2.docx]

Online Supplementary Material


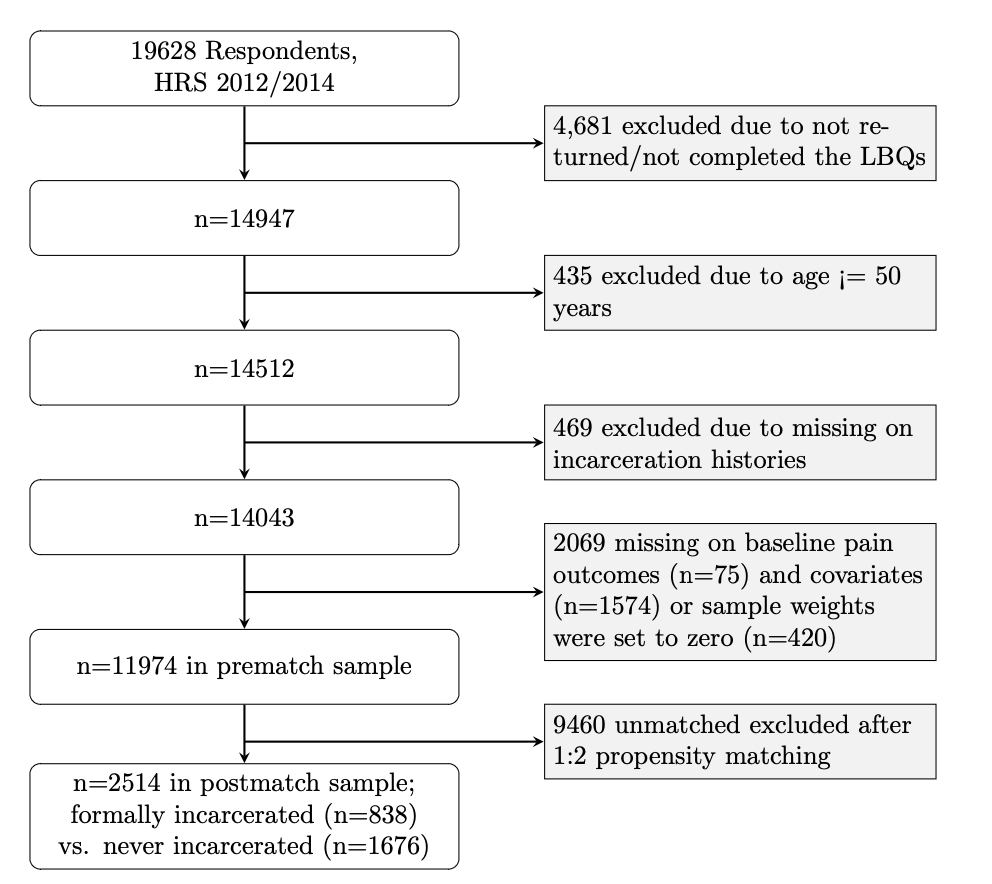


**eFigure 1**. Flow chart displaying assembly of matched cohort.


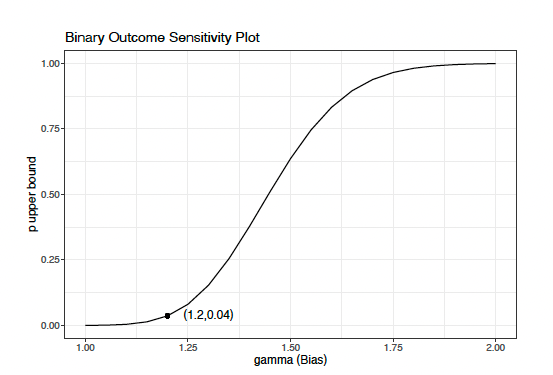


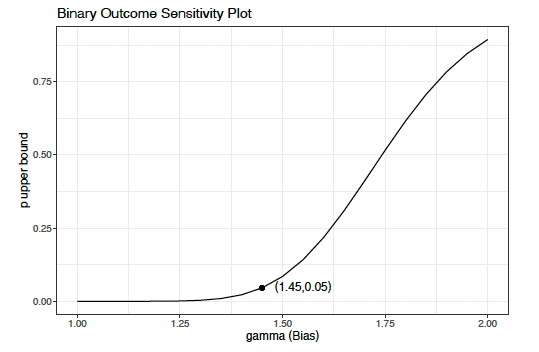


**eFigure 2.** Sensitivity Plots of Gamma Statistics for Binary Outcomes (Upper: Moderate-to-Severe Pain; Lower: Pain-Related Limitation)

**eTable 1.** Weighted Generalized Estimating Equations (WGEE) Models of Pain Outcomes using Pre-matching Full Sample (HRS 2012-2018; n=11,974)

|  | Moderate-to-severe Pain | |  | Pain with physical limitations | |
| --- | --- | --- | --- | --- | --- |
|  |  |  |  |  |  |
|  | PR | 95% CI |  | PR | 95% CI |
| Incarcerated | 1.37*** | 1.24, 1.52 |  | 1.48*** | 1.05, 1.44 |
| Female | 1.29* | 1.21, 1.38 |  | 1.30 *** | 0.87, 1.51 |
| NH Black | 0.91* | 0.83, 0.98 |  | 0.94 | 0.86, 1.03 |
| Hispanic/Latino/a | 0.96 | 0.86, 1.06 |  | 0.95 | 0.85, 1.06 |
| NH other | 1.04 | 0.88. 1.23 |  | 1.03 | 0.86, 1.24 |

**** p <0.001, ** p<0.01, * p <0.05, † p<0.10.*

Notes: All models are controlled for age, race/ethnicity, gender/sex, education, marital status, early life conditions, and adverse childhood experience, and time fixed effects. All models fit weighted generalized estimating equations (WGEE).

**eTable 2.** Weighted Generalized Estimating Equations (WGEE) Models of Moderate-to-Severe Pain and Pain with Physical Limitation (HRS 2012-2018; n=1678)

|  | Moderate-to-Severe Pain | |  | Pain with Physical Limitation | |
| --- | --- | --- | --- | --- | --- |
|  |  |  |  |  |  |
|  | PR | 95% CI |  | PR | 95% CI |
| Ever Incarcerated | 1.27** | 1.10, 1.46 |  | 1.37*** | 1.18, 1.59 |
| Female (ref. male) | 1.39** | 1.18, 1.63 |  | 1.37** | 1,15, 1.63 |
| Race/ethnicity (ref. NH white) |  |  |  |  |  |
| NH Black | 0.99 | 0.84, 1.15 |  | 1.00 | 0.84, 1.20 |
| Hispanic/Latino/a | 0.96 | 0.76, 1.21 |  | 1.04 | 0.83, 1.32 |
| NH other | 1.38† | 1.00, 1.91 |  | 1.51* | 1.08, 2.10 |

**** p <0.001, ** p<0.01, * p <0.05, † p<0.10*

Notes: All models are controlled for trouble with police before age 18 and time fixed effects. All models fit weighted generalized estimating equations (WGEE).
